# Thoracoscopic infrared ablation to create a box lesion as a treatment for atrial fibrillation

**DOI:** 10.1186/s13019-021-01750-1

**Published:** 2022-01-08

**Authors:** Hiroshi Kubota, Toshiya Ohtsuka, Mikio Ninomiya, Takahiro Nonaka, Motoyuki Hisagi, Hidehito Endo, Sachito Minegishi, Hiroshi Tsuchiya, Yusuke Inaba

**Affiliations:** 1 Department of Cardiovascular Surgery, Kyorin University, 6-20-2, Shinkawa,Tokyo, Mitaka 181-8611 Japan; 2 Department of Cardiac Surgery, New Heart Watanabe Institute, Tokyo, Japan; 3 Department of Cardiovascular Surgery, Tokyo Metropolitan Tama Medical Center, Tokyo, Japan

**Keywords:** Atrial fibrillation, Ablation, Infrared, Infrared coagulator, Epicardial ablation, Epicardial maze procedure, Ex-maze procedure, Box lesion, Thoracoscopic surgery, Left atrial appendage amputation

## Abstract

**Background:**

Creating a box lesion in the posterior wall of the left atrium from the epicardial side of the beating heart remains a challenge. Although a transmural lesion can be created by applying radiofrequency (RF) energy at clampable sites, it is still difficult to create a transmural lesion at unclampable sites because the inner blood flow in the unclampable free wall weakens the thermal effect on the outside. Our aim was to apply the newly developed infrared coagulator to create linear transmural lesions on the beating heart thoracoscopically to treat atrial fibrillation (AF).

**Case presentation:**

A 71-year-old male was referred to our hospital with a diagnosis of hypertrophic cardiomyopathy and permanent atrial fibrillation. The patient was first diagnosed with atrial fibrillation 20 years before. Direct current cardioversion had been performed every few years a total of four times, but sinus rhythm restoration had always been temporary. On February 27, 2020, thoracoscopic PV isolation together with infrared roof- and bottom-line ablation to create a box lesion and left atrial appendage amputation (LAAA) were performed. The coagulator could be applied to clinical thoracoscopic surgery to successfully create a box lesion without any complication. The patient restored a regular sinus rhythm, it has been maintained for eleven months, and there have been no adverse events.

**Conclusions:**

The infrared coagulator might have enough potential to create transmural lesions on the beating heart in thoracoscopic AF surgery.

**Supplementary Information:**

The online version contains supplementary material available at 10.1186/s13019-021-01750-1.

## Background

AF is the most common arrhythmia in clinical setting. Conversion to regular sinus rhythm through catheter based approaches and surgically based approaches has been reported with improved outcomes [1]. The hybrid convergent ablation begets endocardial pulmonary vein isolation, epicardial left atrial posterior wall isolation, so called box lesion, and left atrial appendage ligation clinically [2].

However, creating a box lesion in the posterior wall of the left atrium from the epicardial side of the beating heart remains a challenge. Although a transmural lesion can be created by applying RF energy at clampable sites [3], it is still difficult to create a transmural lesion at unclampable sites because the inner blood flow in the unclampable free wall weakens the thermal effect on the outside. because the inner blood flow in the unclampable free wall weakens the thermal effect on the outside. Our aim was to apply the newly developed infrared coagulator to create linear transmural lesions on the beating heart thoracoscopically to treat atrial AF.

## Technology

**Fig. 1:**
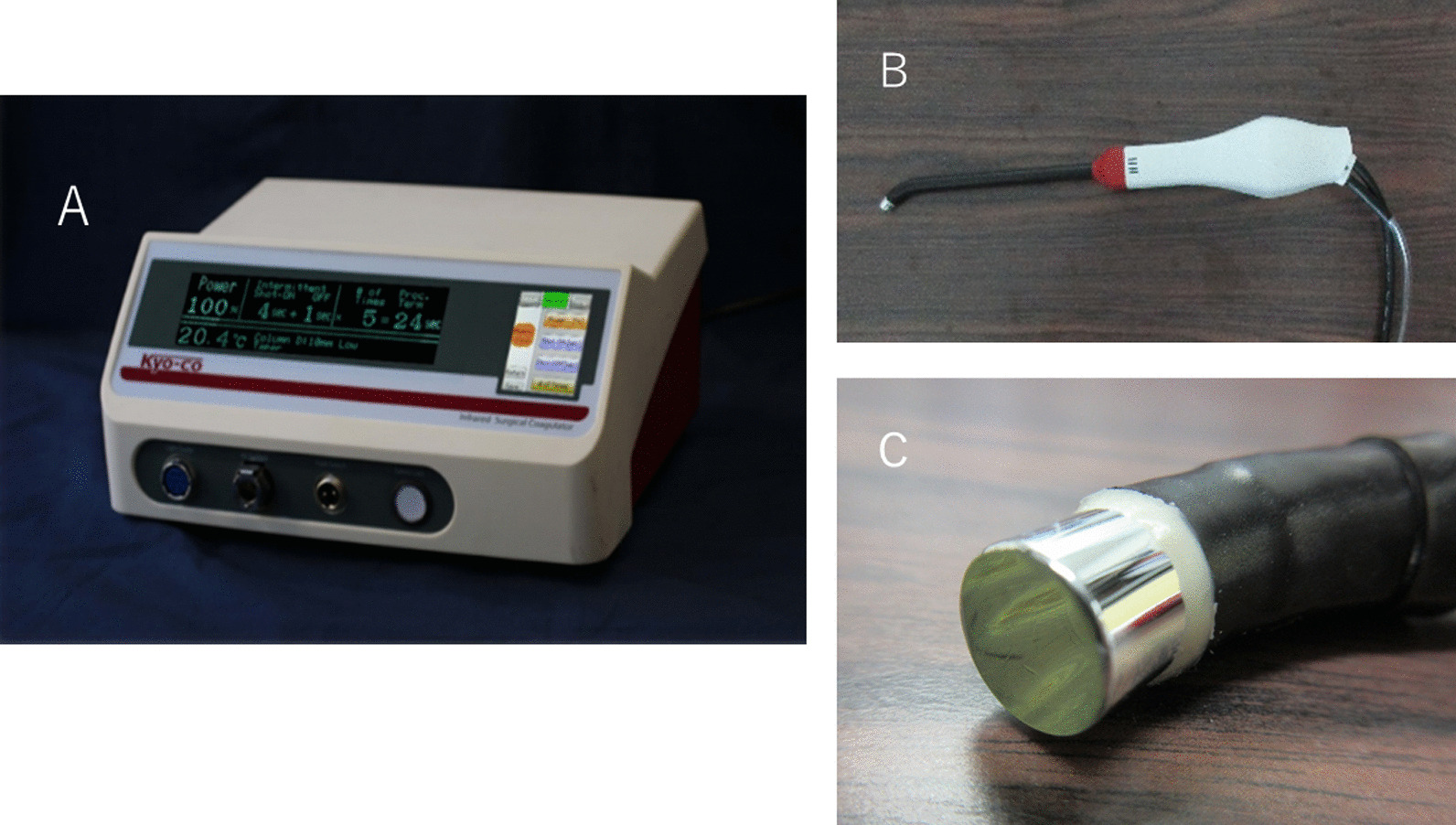
Infrared coagulator “Kyo-co”. **a** Body of the coagulator. It is connected to a light-guide, foot switch, and vacuum system to cool the light-guide. **b** Light-guide reinforced with heat-resistant plastic equipped with a light conducting 8-mm diameter curved-tip quartz rod. **c** Exit plane

The newly developed infrared coagulator is called the “Kyo-co (Photon Co., Ltd., Saitama, Japan).” A reflector focuses light from a tungsten-halogen lamp into a light conducting 8-mm diameter curved-tip quartz rod, and it emerges as 35 W/cm [2] of near-infrared energy (wavelength, 400 to 1,600 nm; peak wavelength, 850 nm). Power output, ablation time, interval time, and total number of applications can be adjusted as needed, and maximum ablation time is limited to 90 s. The distal exit-plane of the light-conducting rod has a round surface (Fig. 1a-c).

## Case presentation

A 71-year-old male was referred to our hospital (Tokyo Metropolitan Tama Medical Center) with a diagnosis of hypertrophic cardiomyopathy and permanent atrial fibrillation. The patient was first diagnosed with atrial fibrillation 20 years before. Direct current cardioversion had been performed every few years a total of four times, but sinus rhythm restoration had always been temporary. The patient’s CHADS2 score was 0. Although he was being treated with edoxaban tosilate monohydrate, he wanted to be free from palpitations, and fear of cerebral infarction. Preoperative transthoracic echography revealed a left ventricular ejection fraction of 65% and left atrial diameter of 56 mm, and there was no valvular dysfunction. Cardiac scintigraphy showed no evidence of myocardial ischemia. The electrocardiogram indicated AF with a 0.2 mV of f-wave amplitude.

On February 27, 2020, thoracoscopic PV isolation together with infrared roof- and bottom-line ablation to create a box lesion and LAAA were performed (Additional file 1: video).

## Operative procedure

**Fig. 2:**
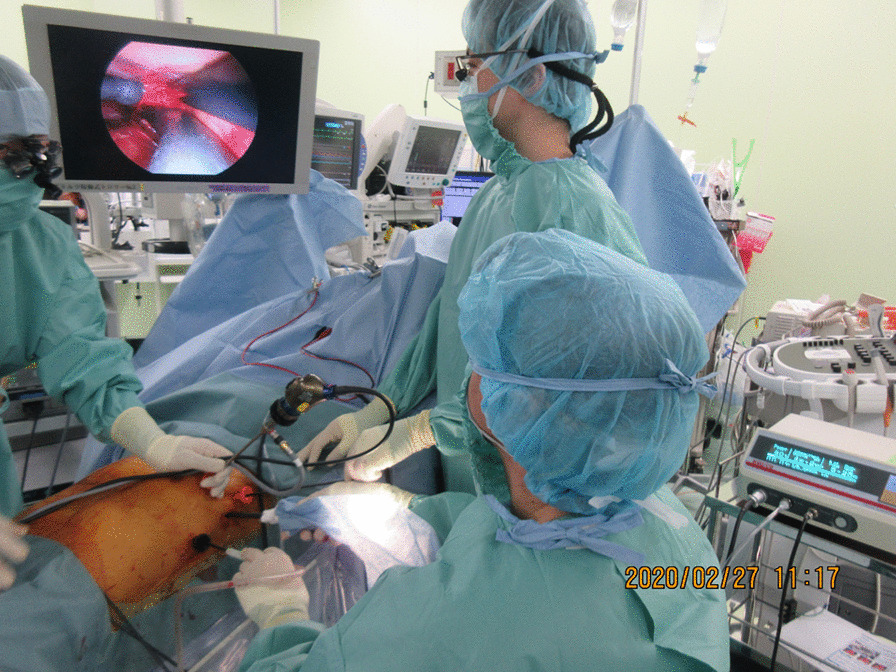
Operating room. Four endoscopic ports were created in the left lateral thorax: one for a 5-mm, 45-degree endoscope, another for an endoscopic cutter, and the other two for endo-forceps

In 2014, the ethics committee of Kyorin University approved a clinical and epidemiologic study entitled, Surgical treatment of arrhythmias, infectious endocarditis, infected aortic aneurysms, and cardiac tumors with an infrared coagulator (Reference number: H26-048). Written consent was obtained from the patient. The patient was anesthetized through a double-lumen endotracheal tube and placed in the supine position. Transesophageal echocardiography (TEE) was performed with the patient in the optimal position to display the left atrial appendage (LAA). The left lung was allowed to deflate, and 4 endoscopic ports were created in the left lateral thorax: one for a 5-mm, 45-degree camera, another for an endoscopic cutter (EZ45G Endoscopic Linear Cutter, Ethicon Endo-Surgery, Cincinnati, Ohio), and the other two for endo-forceps. A 5-cm-long pericardiotomy was made just above the LAA and 2 cm anterior and parallel to the left phrenic nerve, and the ligament of Marshall was sharply divided. An endoscopic light-guided dissector allowed the attached tape to encircle the PVs to enable introduction of the left AtriCure clamp coagulator. The clamp coagulator was then used to cross-clamp the left atrium and thereby isolate the left PVs by means of four times of RF ablations. Finally, an Endostapler (ECHELON FLEX™ powered ENDOPATH™ Stapler 60, ETHICON) was used to clamp and amputate the LAA at its root with a single cartridge. Complete hemostasis was achieved. A TEE examination confirmed complete closure of the LAA. An Endoloop ligature was passed around the corner of the cut edge of the LAA, and it was ligated. Then, a Kyo-co infrared coagulator was inserted and applied to the roof and bottom of the left atrium to produce overlapping linear lesions on the left side under the following set of conditions: 85% power, 4 s of ablation, 2 s interval, and 5 applications (Fig. 2).

**Fig. 3:**
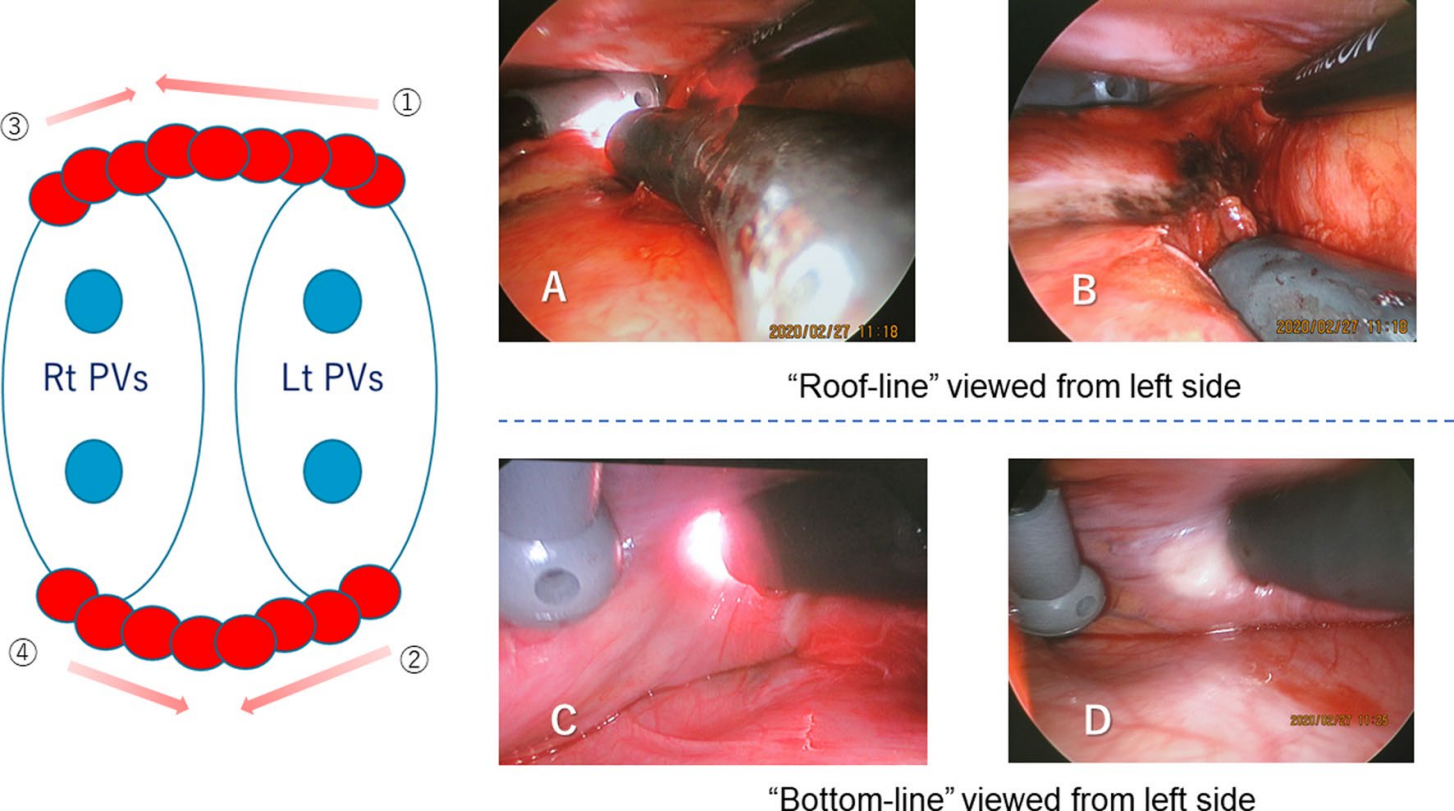
Endoscopic view of the left atrium. **a** Ablation of the left side of the roof of the left atrium. Several overlapping lesions were created to form a linear lesion. An ablated discolored lesion is clearly visible. **b** Ablation of the left side of the bottom of the left atrium. A wide, clear visual field can be obtained with a 45-degree endoscope

After completing the procedure on the left side, the operator moved to the right side of the patient, port placements similar to those on the left were made. The connective tissue between the left atrium and right pulmonary artery, and then below the inferior PV was bluntly dissected. The right clamp coagulator was used to achieve four PV isolations, and then the superior vena cava was isolated 5 mm from the right atrium. The Kyo-co infrared coagulator was inserted and applied to the roof and bottom of the left atrium to produce overlapping linear lesions on the right side (Fig. 3).

The operation time was 90 min. The patient was extubated in the operating room, and he was discharged uneventfully on postoperative day 6. Because he was suffered from hypertrophic cardiomyopathy, treatment with edoxaban tosilate monohydrate was continued and amiodarone was administered. Eleven months after the operation, the patient is well; a regular sinus rhythm which was confirmed by electrocardiograms recorded at each outpatient care has been maintained, and there have been no adverse events.

## Discussion and conclusions

To manage conventional treatment-refractory persistent AF patients, many catheter based and surgery based approach has been reported. Wats et al. reviewed a cumulative experience in over 10,000 patients, and concluded that the convergent hybrid procedure now has an established position in the vast array of procedures directed at managing non-paroxysmal AF [4]. However, although several devices capable of creation a box lesion in the left atrium by an epicardial approach have been reported, there is still no reliable device available that can be used to create a transmural lesion on the beating heart.

Bulava et al. assessed the efficacy of epicardially created lesions produced with bipolar RF energy in 70 patients who had persistent AF, and they reported success rates for creating a conduction block across the bottom line and roof line connecting the two inferior and superior PVs of only 58.0% and 24.3%, respectively [5]. Nath et al. demonstrated that hyperthermia induced by RF energy causes significant changes in the electrophysiological properties of myocardiocytes. They observed reversible loss of cellular excitability after exposure to temperatures in the 42.7℃ to 51.3℃ range (median, 48.0℃) for 60 s and irreversible loss of cellular excitability and tissue injury after exposure to temperatures > 50℃ for 60 s [6]. Our experiment in which we exposed chicken skeletal muscle to infrared energy showed that tissue temperature rose to a maximum of 97.9 ± 2.1℃ for a total of 28 s (4 s × 5 exposures at 2-s intervals). Thus, theoretically, infrared energy has a more reliable ability to create a transmural lesion on a warm, beating atrium than cryothermia does. Further, because the maximum tissue temperature is far higher than fat tissue melting point, when we apply the device, the epicardial fat tissue melts within few seconds so that the fat tissue does not disturb the thermal effect on the myocardium.

We previously reported the results of series of animal model experiments demonstrating that infrared energy enables creation of a transmural lesion on the canine beating right ventricle to a maximum depth of 10.3 mm, and creation of a conduction block on a beating right atrium [7–10]. Successful electrical isolation of the right atrial appendage has been confirmed in a clinical setting [11]. Moreover, using a 30 × 10 mm of cuboid shaped conducting rod, by confirming the prolongation of conduction time between two electrodes put at both side of the probe, irreversible conduction block could be confirmed on a beating right atrial free wall, and this finding showed that an “electrophysiological transmural lesion” as well as “histopathological transmural lesion” could be created on the beating atrium by exposing it to infrared energy [12].

Ohtsuka et al. have reported the efficacy of thoracoscopic LAAA in preventing cerebral infarctions [13]. Our new approach, which enables rhythm control, adds value to simple LAAA for AF patients.

No electrophysiological studies were performed on our patient, however, because direct-current-cardioversion-resistant chronic AF was converted to regular sinus rhythm and has been maintained, it might be supporting evidence to prove the effectiveness of our new device. After the successful treatment of permanent AF by thoracoscopic epicardial infrared ablation in our patient, 50 consecutive cases of AF have been treated in the same procedure safely without any complication.

In conclusion, the infrared coagulator might have enough potential to create transmural lesions on the beating heart in clinical thoracoscopic AF surgery concomitant with LAAA.

## Supplementary Information

**Additional file 1.** Thoracoscopic infrared ablation to create roof and bottom lesions of the left atrium.

## Data Availability

Supporting data are available upon request to the corresponding author. Publication of raw data is impossible as it would conflict with our privacy policy.

## Abbreviations

AF: Atrial fibrillation
LAA: Left atrial appendage
LAAA: Left atrial appendage amputation
PV: Pulmonary vein
RF: Radiofrequency
TEE: Transesophageal echography

## References

[1] Calkins H, Hindricks G, Cappato R, Kim YH, Saad EB, Aguinaga L (2017). 2017 HRS/EHRA/ECAS/APHRS/SOLAECE expert consensus statement on catheter and surgical ablation of atrial fibrillation. Heart Rhythm.

[2] Umbrain V, Verborgh C, Chierchia GB, de Asmundis C, Brugada P, Meir M (2017). One-stage approach for hybrid atrial fibrillation treatment. Arrhythm Electrophysiol Rev..

[3] Doty JR, Clayson SE (2012). Surgical treatment of isolated (lone) atrial fibrillation with Gemini-S Ablation and Left Atrial Appendage Excision (GALAXY procedure). Innovations.

[4] Wats K, Kiser A, Makati K, Sood N, DeLurgio D, Greenberg Y (2020). The convergent atrial fibrillation ablation procedure: evolution of a multidisciplinary approach to atrial fibrillation management. Arrhythm Electrophysiol Rev.

[5] Bulava A, Mokracek A, Kurfirst V (2017). Delayed electroanatomic mapping after surgical ablation for persistent atrial fibrillation. Ann Thorac Surg.

[6] Nath S, Lynch C, Whayne JG, Haines DE (1993). Cellular electriphysiological effcts of hyperthermia on isolated Guinea pig papillary muscle. Implic Catheter Ablation Circ.

[7] Kubota H, Furuse A, Takeshita M, Kotsuka Y, Takamoto S (1998). Atrial ablation with an IRK-151 infrared coagulator. Ann Thorac Surg.

[8] Kubota H, Takamoto S, Takeshita M, Miyaji K, Kotsuka Y, Furuse A (2000). Atrial ablation using an IRK-151 infrared coagulator in canine model. J Cardiovasc Surg.

[9] Kubota H, Takamoto S, Furuse A, Sato M, Endo H, Fujiki T (2005). Epicardial maze procedure on the beating heart with an infrared coagulator. Ann Thorac Surg.

[10] Kubota H, Kenichi S, Takamoto S, Endo H, Tsuchiya H, Yoshimoto A, Choi JI (2011). Clinical result of epicardial pulmonary vein isolation (LAVIE) by cryoablation as concomitant cardiac operation and clinical application of new device (KIRC-119 infrared coagulator) to treat atrial fibrillation. Atrial fibrillation-basic research and clinical applications.

[11] Kubota H, Sudo K, Takamoto S, Tonari K, Fujiki T, Endo H (2009). Epicardial electrical isolation of the right atrial appendage on the beating heart with an infrared coagulator. Ann Thorac Surg.

[12] Kubota H, Endo H, Ishii H, Tsuchiya H, Inaba Y, Takahashi Y (2018). Epicardial infrared ablation to create a linear conduction block on a beating right atrium. J Cardiothorac Surg.

[13] Ohtsuka T, Ninomiya M, Nonaka T, Hisagi M, Ota T, Mizutani T (2013). Thoracoscopic stand-alone left atrial appendectomy for thromboembolism prevention in nonvalvular atrial fibrillation. J Am Coll Cardiol.

